# Comparing approaches to ordering peanut component–resolved diagnostics to reduce the need for oral food challenges

**DOI:** 10.1016/j.jacig.2025.100440

**Published:** 2025-02-15

**Authors:** Raymond Mak, Edmond S. Chan, Michael Irvine, Jia Yi Huang, James Hethey, Sheila Hartstein, Li Wang

**Affiliations:** a Department of Pediatrics, Division of Allergy, British Columbia Children’s Hospital, University of British Columbia, Vancouver, British Columbia, Canada; b British Columbia Children’s Hospital Research Institute, Vancouver, British Columbia, Canada; c British Columbia Center for Disease Control, Faculty of Health Sciences, Simon Fraser University, Burnaby, British Columbia, Canada; d Faculty of Medicine, University of British Columbia, Vancouver, British Columbia, Canada; e Department of Pathology and Laboratory Medicine, University of British Columbia, Vancouver, British Columbia, Canada

**Keywords:** Peanut allergy, component-resolved diagnostic testing, oral food challenges

## Abstract

**Background:**

Peanut component–resolved diagnostics (peanut CRD) is a potentially valuable tool for distinguishing between anaphylactic peanut allergies and milder phenotypes, such as pollen–food allergy syndrome. However, the optimal strategy for integrating CRD into clinical practice remains unclear.

**Objective:**

This study aims to evaluate the rates of oral food challenge (OFC) when CRD is ordered: routinely for all patients, selectively on the basis of clinical characteristics, or guided by other peanut biomarkers.

**Methods:**

We compared OFC rates between 2 cohorts. Cohort 1 included patients with peanut allergy who received CRD as part of routine testing, regardless of clinical features. In cohort 2, CRD was ordered selectively, depending on factors such as older age, comorbidities, or pollen sensitization. OFC was offered at the physician's discretion in both cohorts. Later, a proposed 2-step clinical algorithm was retrospectively applied to the pooled data to determine patients eligible for OFC.

**Results:**

A total of 322 patients (137 in cohort 1 and 185 in cohort 2) participated in the study. OFC rates were lower in the selective testing group (9.7%) and with use of an algorithm (9.5%) than in the group that underwent routine testing (25.5%). The correlation between peanut-specific IgE level and CRD result was high (*R* = 0.85).

**Conclusion:**

Offering CRD selectively on the basis of clinical characteristics and being guided by a 2-step algorithm are more efficient strategies that can reduce rates of OFC to enhance patient safety, optimize health care resource utilization, and reduce costs. As peanut-specific IgE level and CRD result correlate well, testing for Ara h 2 is likely redundant at very high and low ranges.

## Introduction

Peanut component–resolved diagnostics (peanut CRD) testing may be a valuable tool for older, pollen-sensitized children with remote clinical histories, helping to differentiate anaphylactic phenotypes from pollen–food allergy syndrome.^1^ However, whether clinicians should routinely order peanut CRD in all patients, selectively order CRD on the basis of clinical characteristics, or order CRD on the basis of other peanut biomarkers remains unknown. Our goal was to assess the rate of oral food challenge (OFC) with each approach.

Others have previously proposed diagnostic algorithms using testing for peanut-specific IgE (peanut sIgE) level or skin prick testing (SPT) as initial tests, followed by CRD if the initial results are within a predetermined range.^2^ For cashew, Brettig et al showed that using a 2-step algorithm starting with testing for cashew sIgE followed by testing for Ana o 3 resulted in substantially fewer OFCs (in 11.4% of participants) compared with SPT or testing for sIgE (58.4%-74.6%) alone.^3^ However, we are not aware of similar studies for peanut.

**Fig 1:**
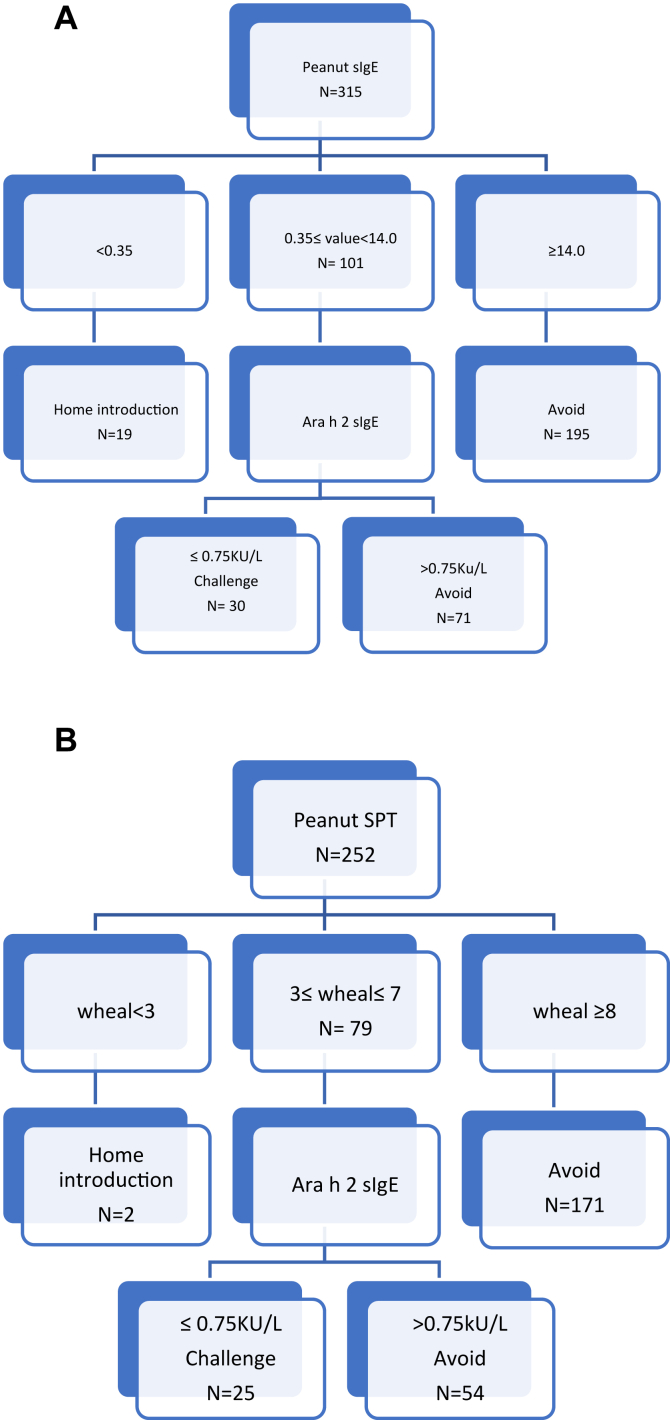
Proposed 2-step algorithms for using CRD with peanut sIgE (**A**) and skin testing (**B**).

We propose 2-step algorithms based on initial peanut skin test or peanut sIgE test results, followed by CRD to assess the hypothetical rates of OFC using our pooled cohort data. Our algorithm (Fig 1) is based on previous studies demonstrating that an average wheal size of at least 8 mm and a peanut sIgE level of at least 14 kU/L have a 95% positive predictive value for peanut allergy.^4^ Our center previously reported that an Ara h 2 sIgE level higher than 0.75 kU/L was able to predict the OFC outcome correctly 75% of the time.^5^

Ethics approval was obtained from the University of British Columbia research ethics board. Patients with a known or suspected peanut allergy from a single tertiary pediatric allergy clinic in British Columbia Children’s Hospital were included.

In the case of cohort 1, the members of which were enrolled between November 2011 and March 2013, any patient with a suspected diagnosis of peanut allergy was eligible to receive CRD as part of routine testing regardless of clinical characteristics. In the case of cohort 2, the members of which were enrolled between June 2018 and November 2022, patients received “selective CRD testing” when their physician thought that it was clinically indicated on the basis of clinical characteristics, which included older age, presence of comorbidities (eczema or symptomatic rhinitis), or pollen sensitization determined by skin testing. For both cohorts, OFC was offered at the physician's discretion.

With retrospective application of the proposed 2-step clinical algorithms (Fig 1) to our pooled cohort data, patients were deemed eligible for OFC if either (1) they had a peanut sIGE level of at least 0.35 kU/L but less than 14.0 kU/L *and* an Ara h 2 level not exceeding 0.75 kU/L or (2) skin testing determined that they had wheal size of at least 3 mm but not exceeding 7 mm *and* an Ara h 2 level not exceeding.75 kU/L.

Data were obtained via retrospective chart review. The most recent CRD result was extracted for each patient. SPT results were extracted for each patient from the test closest in time to the most recent CRD result. Age was derived from the difference between the patient’s date of birth and the most recent CRD result. Using our hospital’s laboratory database, we estimated the relative test utilization rate of CRD testing versus that of testing for peanut sIgE that were ordered during the study of cohort 2.

Our primary outcome was the rate of OFC offered between each strategy. Secondary outcomes included a correlation analysis between levels of peanut sIgE and Ara h 2 and OFC outcomes. The strength of correlation between sIgE and Ara h 2 was estimated by using the Pearson correlation coefficient. All analyses were conducted using R, version 4.3.1.

## Results and discussion

A total of 322 patients (137 patients in cohort 1 and 185 in cohort 2) were included in our study. The demographics and clinical characteristics of cohorts 1 and 2 are outlined in Table I. In the case of cohort 2, children were on average older (*P* < .001**)**, had higher rates of rhinitis (*P* < .01) and eczema (*P* < .001), and were more likely to have a history of anaphylaxis in response to peanut (*P* = .033). Compared with cohort 1, cohort 2 had a lower peanut sIgE (*P* = .0040) and median Ara h 2 (*P* = .02) levels. During the selective ordering phase, there was a 7.6% rate of CRD utilization relative to rate of peanut sIgE testing (185 of 2443 tests).

**Table I:** Demographic and clinical variables in cohorts 1 and 2 (N = 322)

Characteristic	Cohort 1	Cohort 2
Cohort size	137	185
Previous reaction to peanut, no. (%)	71 (61.2%)	125 (73.5%)
Age at time of CRD (y), mean (SD)	8.40 (3.62)	11.6 (3.52)
History of anaphylaxis, no. (%)	20 (17.2%)	49 (29.0%)
Sex, no. (%)	Male: 90 (65.7%)	Male: 139 (58.4%)
	Female: 47 (34.3%)	Female: 46 (41.3%)
Comorbidities, no. (%)	Eczema: 85 (63.9%)	Eczema: 155 (85.6%)
	Asthma: 60 (45.1%)	Asthma: 94 (51.9%)
	Rhinitis: 77 (58.3%)	Rhinitis: 132 (72.9%)
Aeroallergen sensitization, no. (%)	Tree: 68 (58.6%)	Tree: 109 (63.0%)
	Grass: 83 (70.9%)	Grass: 126 (73.7%)
Peanut immunotherapy, no. (%)	0 (0.0%)	12 (6.6%), including 8 who received oral immunotherapy and 4 who received sublingual immunotherapy
Skin test wheal size (mm), mean (SD)	8.62 (2.90)	8.96 (3.38)
sIgE level quantitation (ug/L), mean (SD)	50.71 (40.51)	40.92 (41.65)
sIgE level quantitation (ug/L), median (IQR)	45.20 (6.66-101.00)	21.20 (2.79-93.20)
Ara h 2 level quantitation (kU/L), mean (SD)	42.77 (39.80)	34.45 (39.31)
Ara h 2 level quantitation (kU/L), median (IQR)	34.30 (2.54-83.90)	12.39 (1.54-67.76)
Ara h8 quantitation (kU/L), no. (SD)	3.71 (10.16)	9.15 (19.92)
Ara h8 quantitation (kU/L), median (IQR)	0.01 (0.00-0.26)	0.28 (0.00-6.72)
OFC (n = 53)	Total 35, including 20 who failed (57%) and 15 who passed (42.9%)	Total 18, including 8 who failed (44.4%) and 10 who passed (55.6%)
Epinephrine use during OFC, no. (%)	10 (50%)	1 (12.5%)

*IQR*, Interquartile range.

**Fig 2:**
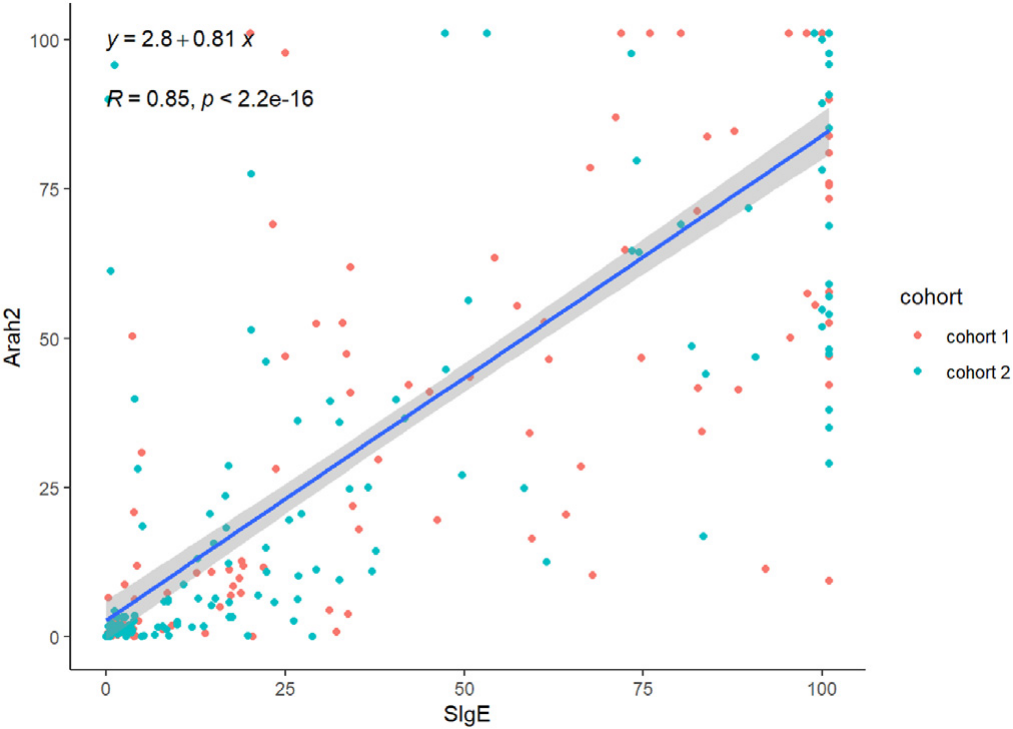
Correlation analysis of peanut IgE and Ara h 2 levels.

In cohort 1, OFC was performed on 25.5% of patients (35 of 137), whereas only 9.7% of patients in cohort 2 (18 of 185) received an OFC (*P* = .0002). The OFC pass rates were similar in cohort 2 (55.6%) and cohort 1 (42.9%), but epinephrine use was lower in cohort 2 (used in 12.5% vs 50% of cohort members), although the difference was not statistically significant (*P* = .0159). With use of a 2-step algorithm starting with sIgE level, 30 of 315 patients (9.5%) would be eligible for OFC (Fig 1). The data for those 30 individuals eligible for OFC are incomplete, as not all eligible candidates received OFC; however, 11 of 18 OFCs (61%) were successful. Our data show a high degree of correlation between peanut and Ara h 2 sIgE levels (*R* = 0.85) (Fig 2).

A limitation of our study is that during the study of cohort 2, clinicians ordered CRD without strictly predetermined clinical criteria, leading to variability in practice, whereby physicians may emphasize different clinical characteristics when ordering CRD. How truly selective the physicians were is unknown, as the total number of patients with peanut allergy during the study of cohort 2 was likely less than the number of sIgE tests ordered. Using the number of peanut sIgE level tests run by our laboratory as a surrogate is an imperfect estimate of selectivity.

Despite these limitations, the use of a “selective ordering approach” or a 2-step algorithm in cohort 2 resulted in significantly fewer OFCs than in cohort 1. Of relevance, 12 patients in cohort 2 received food immunotherapy, whereas no patients in cohort 1 received immunotherapy. However, the presence of food immunotherapy did not significantly skew either the number of OFCs or the success rate. OFC safety as assessed by epinephrine use was potentially better for cohort 2. As OFC is time-consuming, carries a risk of anaphylaxis (and rarely even a risk of fatality), offering OFC to those with the best chance of success would carry an advantage in terms of increased patient safety and appropriate utilization of health care resources.

The 2-step algorithm has the added benefit of fewer total CRD tests ordered and cost savings. A previous study in 2020 showed that the average cost of an OFC in Canada was $172.05 compared with $13.40 for sIgE testing.^6^ In 2019, the cost incurred by our laboratory to send IgE to Ara h 2 and Ara h 8 outside the province was $120. Although the cost for CRD has decreased to a combined cost of $65.68 in 2024, it remains almost 5 times as costly as peanut sIgE testing. Given the high degree of correlation, at very high and low levels of peanut sIgE, ordering Ara h 2 testing is likely redundant. With use of the algorithm, only 101 patients would have received CRD testing. Although the OFC pass rate for this strategy was incomplete, the data that we did have were reassuring. Further validation of its diagnostic accuracy is needed to establish its real-world utility.

Currently in Canada, public reimbursement of CRD is province dependent, with access being highly variable between provinces. In British Columbia, CRD is not routinely available. When a test is ordered, payment would be through research funding, private pay, or submission of a funding application for out-of-province testing to the government to justify testing. Because of the greater simplicity, easier implementation, and similar outcomes of CRD, we plan to advocate for public reimbursement of CRD testing using the 2-step algorithm, as current access is limited.

**Clinical implications**

Selectively ordering peanut CRD or using a 2-step algorithm based on other biomarker results can reduce the rate of OFC compared with ordering CRD in all comers.

## Disclosure statement

Supported by a Dr Donald B. Rix Pathology & Laboratory Medicine Research Grant.

Disclosure of potential conflict of interest: R. Mak has been a member of advisory boards, as well as speaker receiving honoraria from Pfizer, Medexus, GSK, Arcutis, CSL Behring, Sanofi, AstraZeneca, ALK, and Novartis. E. S. Chan has received research support from DBV Technologies and has been a member of advisory boards for Pfizer, Miravo, Medexus, Leo Pharma, Kaleo, DBV, AllerGenis, Sanofi, Bausch Health, Avir Pharma, AstraZeneca, ALK, and Alladapt. The rest of the authors declare that they have no relevant conflicts of interest.
